# Estimation of Prestress Force Distribution in the Multi-Strand System of Prestressed Concrete Structures

**DOI:** 10.3390/s150614079

**Published:** 2015-06-24

**Authors:** Keunhee Cho, Sung Yong Park, Jeong-Rae Cho, Sung Tae Kim, Young-Hwan Park

**Affiliations:** Structural Engineering Research Institute, Korea Institute of Civil Engineering and Building Technology, 283 Goyangdae-Ro, Ilsanseo-Gu, Goyang-Si, Gyeonggi-Do 411-712, Korea; E-Mails: kcho@kict.re.kr (K.C.); sypark@kict.re.kr (S.Y.P.); chojr@kict.re.kr (J.-R.C.); esper009@kict.re.kr (S.T.K.)

**Keywords:** optical fiber sensor, elastomagnetic sensor, prestressed concrete, strand

## Abstract

Prestressed concrete (PSC) is one of the most reliable, durable and widely used construction materials, which overcomes the weakness of concrete in tension by the introduction of a prestress force. Smart strands enabling measurement of the prestress force have recently been developed to maintain PSC structures throughout their lifetime. However, the smart strand cannot give a representative indication of the whole prestress force when used in multi-strand systems since each strand sustains a different prestress force. In this paper, the actual distribution of the prestress force in a multi-strand system is examined using elastomagnetic (EM) sensors to develop a method for tracking representative indicators of the prestress force using smart strands.

## 1. Introduction

Prestressing is a technique involving the application of a compressive force or prestress force to a reinforced concrete structure so as to realize high quality long spans by improving the weaknesses of concrete to tension. The compressive force is generally introduced by means of strands. However, the prestressed concrete (PSC) structure may collapse due to the occurrence of excessive prestress forces or corrosion due to voids in the grout. This emphasizes the necessity to observe and adequately maintain the prestress force throughout the lifetime of the structure from its construction to its dismantlement. 

Recently, “smart strands” have been developed to measure the prestress force from the construction stage to the operating stage of the PSC structure. Most of these smart strands measure the strain in the strand by means of a fiber-optic sensor embedded in the core wire. This core wire can be a steel tube [1], a CFRP rod [2], or a GFRP rod [3]. A Fiber Bragg Grating (FBG) sensor is commonly used as an optical sensor but optic fiber is also applied [4]. Apart from the method of replacing the core wire, there is also a method installing FBG sensor on the outside of the steel strand [5] but this has been found to be difficult to apply in practice due to the structure of the multi-strand system.

These smart strands are naturally costlier than common steel strands due to the substantial additional costs brought by the fiber-optic sensor and the need to embed the sensor in the strand. This means that it is economically unaffordable to replace all the strands by smart strands in multi-strand system. In addition, Chandoga and Jaroševič [6] reported that the strands in a multi-strand system do not sustain identical prestress forces. Concretely, these authors found out that the distribution of the prestress force in the 708 strands of 59 multi-strand systems ranged between 78% and 112% of the average prestress force. This distribution is due to the prestressing process which simultaneously adjusts the prestress force of several strands to the target value. This implies the impossibility of assessing the distribution of the prestress force in all the strands of the multi-strand system even if the prestress force is measured by replacing a small portion of the strands by smart strands. Accordingly, it is necessary to provide a rational method enabling evaluation of the distribution of the prestress force in all the strands of the multi-strand system using a limited number of smart strands. 

**Figure 1:**
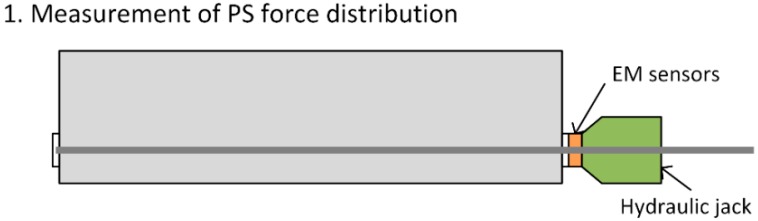

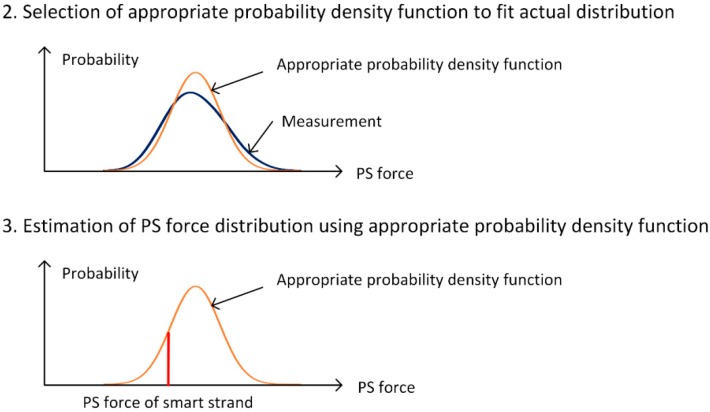
Underlying concept for the evaluation of the prestress force distribution in multi-strand systems.

To that goal, this study intends to assess the distribution characteristics of the prestress force by measuring the prestress force in the multi-strand system and to propose a method for estimating the distribution of the prestress force exploiting the measurements of the smart strands. Figure 1 displays the concept underlying the estimation of the prestress force distribution in the multi-strand system: (1) the actual distribution of the prestress force is first measured using elastomagnetic (EM) sensors; (2) an appropriate probability density function is then selected to fit the measured prestress force distribution; and (3) the distribution and variation of the whole set of prestress forces is estimated based upon the prestress forces measured by the smart strands assuming that the selected distribution function represents the actual prestress force distribution. Since this method enables one to estimate the prestress force distribution all along the lifetime of the PSC structure from its erection to its operating stage, it can be applied for monitoring the structure integrity.

## 2. Experimental Section

**Figure 2:**
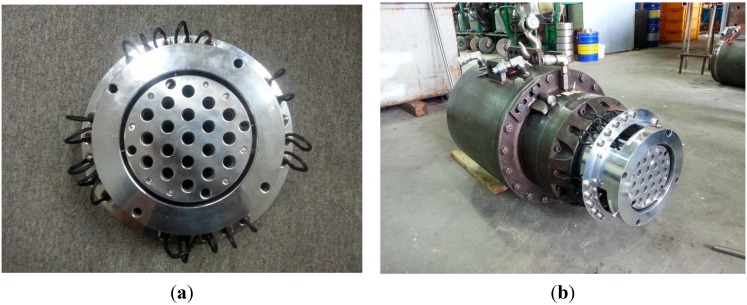
Underlying concept for the evaluation of the prestress force distribution in multi-strand system. (**a**) EM sensor; (**b**) EM sensor attached to hydraulic jack.

Tests were conducted to evaluate the distribution characteristics of the prestress force in the multi-strand system. The elastomagnetic (EM) sensor appeared to be the most suitable sensor for our purpose considering the need to repeatedly measure the prestress force in each individual strand of the multi-strand system to establish a database on the prestress force distribution. The EM sensor is a contactless sensor conceived by noticing the changes in the induced magnetic flux and current crossing the sensor according to the stress state of the material [7]. This sensor is used for the measurement of the resistance load in the stay cables of cable-stayed bridges or in the tendons of PSC structures [8,9]. In this study, the head of the hydraulic jack was fabricated so as to fix the EM sensors and measure the prestress force in the strands (Figure 2).

**Figure 3:**
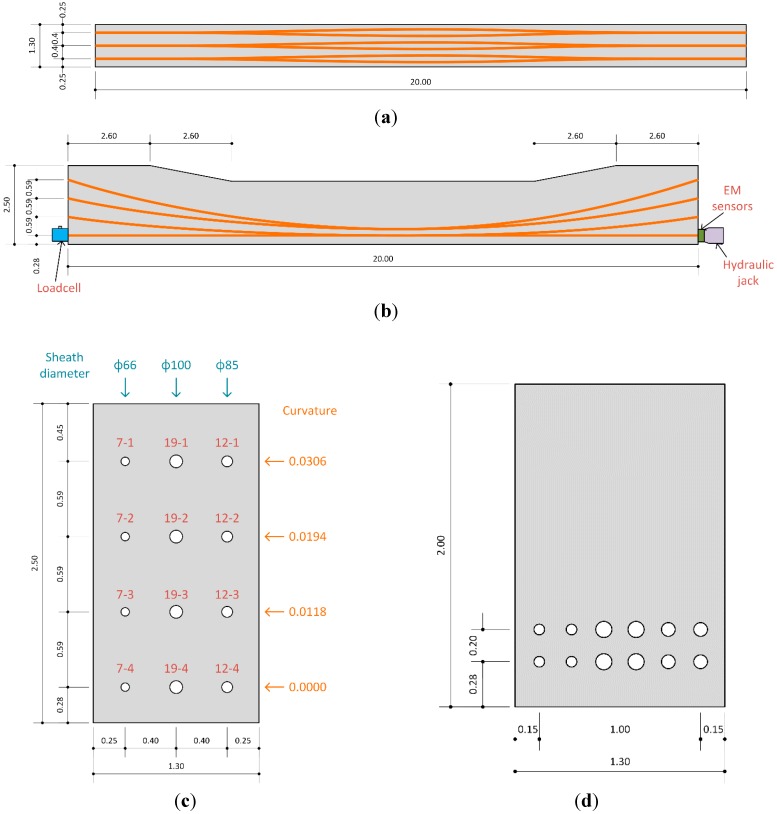
Shape of specimens. (**a**) Elevation; (**b**) Lateral view; (**c**) Cross-section at extremity; (**d**) Cross-section at mid-span.

Specimens were fabricated as shown in Table 1 and Figure 3 to assess the distribution pattern of the prestress force with respect to the number and curvature of the tendons in the anchorage. The twelve anchor heads were installed in the specimen manufactured with lengths of 20 m, heights of 2.0 m to 2.5 m and widths of 1.3 m. Seven-hole, twelve-hole and nineteen-hole anchor heads were utilized and the diameters of the corresponding sheaths were respectively 66, 85 and 100 mm. The sheaths were disposed along the depth in four layers at the extremities and two layers at mid-span, which resulted in a small mid-span transversal deviation. From the highest layer to the lowest, the curvatures were 0.0306 for layer-1, 0.0194 for layer-2 and 0.0118 for layer-3, and 0.0 for layer-4.

**Table 1:** Details of specimens.

Designation	Number of Tendons	Diameter of Sheath (mm)	Curvature (1/m)
7-1	7	66	0.0306
7-2			0.0194
7-3			0.0118
7-4			0.0000
12-1	12	85	0.0306
12-2			0.0194
12-3			0.0118
12-4			0.0000
19-1	19	100	0.0306
19-2			0.0194
19-3			0.0118
19-4			0.0000

**Figure 4:**
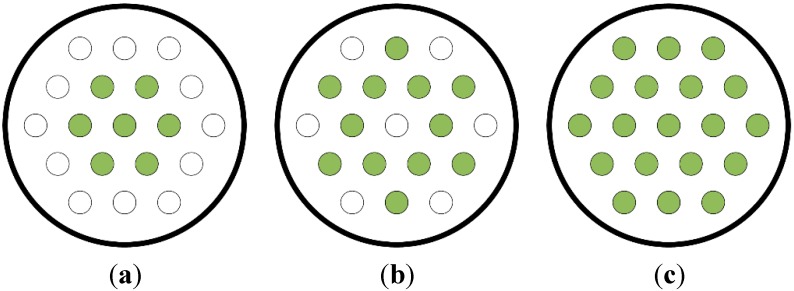
Arrangement of tendons by type of anchor head. (**a**) 7-hole anchor head; (**b**) 12-hole anchor head; (**c**) 19-hole anchor head.

The arrangement of the tendons in the anchor heads is shown in Figure 4. The arrangements in the 7-hole and 19-hole anchor heads correspond to the actual arrangements adopted on site, but the 19-hole anchor head was used as a 12-hole anchor head with the tendons disposed symmetrically.

**Figure 5:**
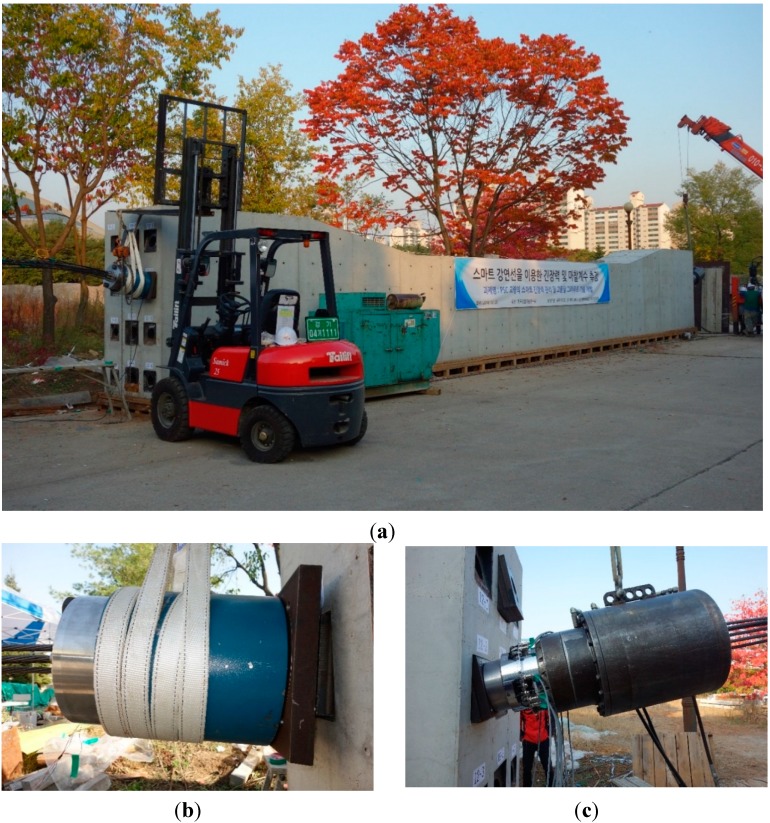
(**a**) View of test; (**b**) Fixed end; (**c**) Prestressed end.

Figure 5 depicts a view of the test. A load cell was installed at the fixed end and prestressing was applied by means of a hydraulic jack equipped with EM sensors attached at its head. Loading was applied to increase the prestress force by 20 kN on the mean in each strand. Note that a larger load was applied at early prestressing for the 7-hole and 12-hole multi-strand systems due to the difficulty in applying relatively small loads at the initial stage. The prestress force of each strand was measured at every loading step using the EM sensors.

## 3. Results

**Figure 6:**
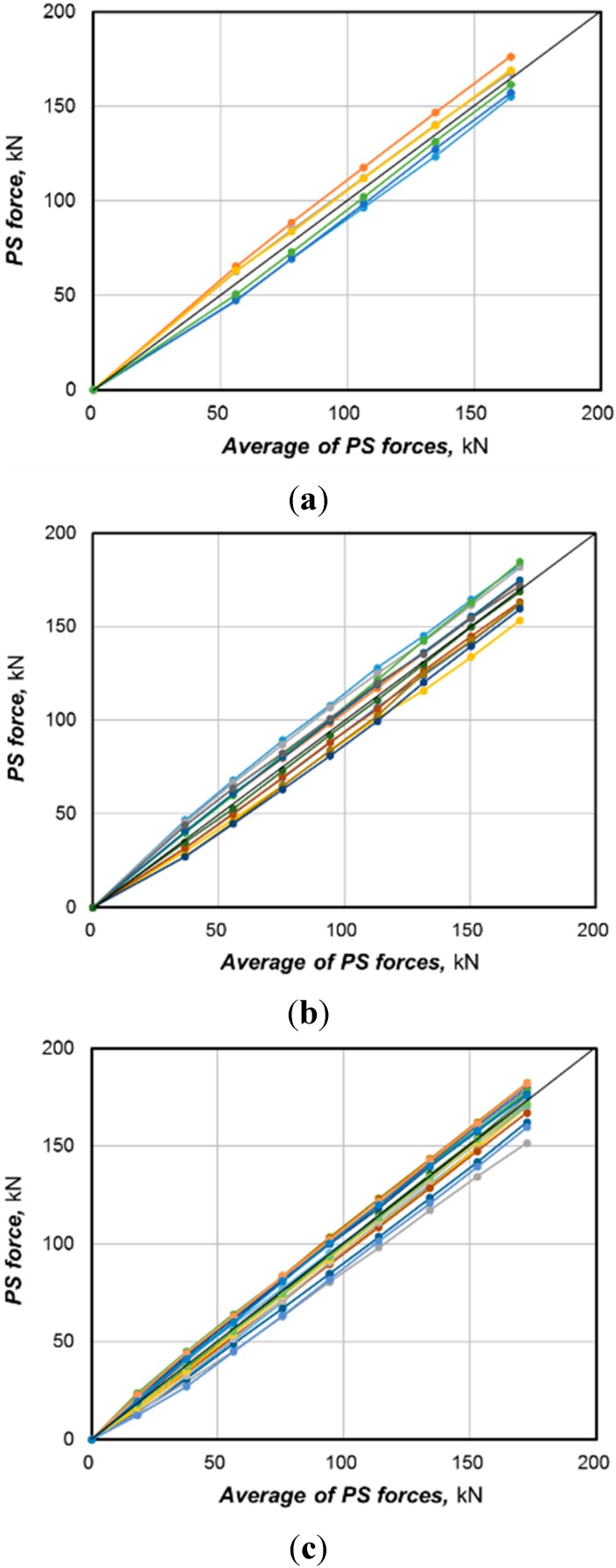
Variation of prestress force according to prestress stage by type of anchor head. (**a**) 7-hole system (specimen 7-1); (**b**) 12-hole system (specimen 12-1); (**c**) 19-hole system (specimen 19-1).

Figure 6 plots the variation of the prestress force measured by the EM sensors at each loading step per type of multi-strand system. The prestress force appears to vary practically linearly with respect to the average prestress force. Similar patterns were also observed for the other specimens. The difference in the prestress force among the strands is seen to occur at early prestressing and increases with larger prestressing but with a relatively reduced rate of increase.

Table 2 lists the average, standard deviation, minimum and maximum values of the prestress force at final prestress for each considered specimen. The standard deviation of the prestress force appears to range between 6.3 kN and 9.7 kN, and the average is 7.8 kN. The minimum and maximum values of the prestress force are 156.7 kN and 184.4 kN, respectively, which correspond to 92% to 108% of the average value of 170.3 kN. This indicates that the distribution of the prestress force is practically symmetric with respect to the average.

**Table 2:** Distribution characteristics of prestress force at final prestress per type of multi-strand system.

Specimen	Number of Holes	Curvature, 1/m	Average Prestress Force, kN	Std. Deviation of Prestress Force, kN	Min. Prestress Force, kN	Min. Prestress Force, kN
7-1	7	0.0306	164.5	7.4	155.0	176.4
7-2	7	0.0194	168.0	6.6	158.8	177.9
7-3	7	0.0118	170.2	8.6	156.7	187.8
7-4	7	0.0000	170.3	8.2	160.1	187.8
12-1	12	0.0306	170.0	9.7	153.5	184.7
12-2	12	0.0194	170.3	9.1	155.4	183.8
12-3	12	0.0118	170.8	6.3	161.2	181.7
12-4	12	0.0000	170.3	6.3	161.1	183.9
19-1	19	0.0306	172.5	7.8	151.5	182.3
19-2	19	0.0194	172.9	7.3	158.7	190.6
19-3	19	0.0118	172.5	9.0	148.6	187.2
19-4	19	0.0000	171.8	7.6	159.2	188.6
Average			170.3	7.8	156.7	184.4

**Figure 7:**
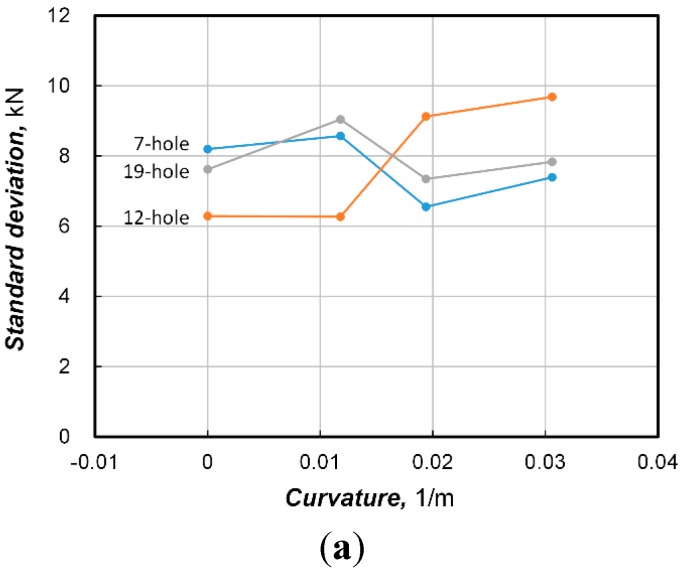

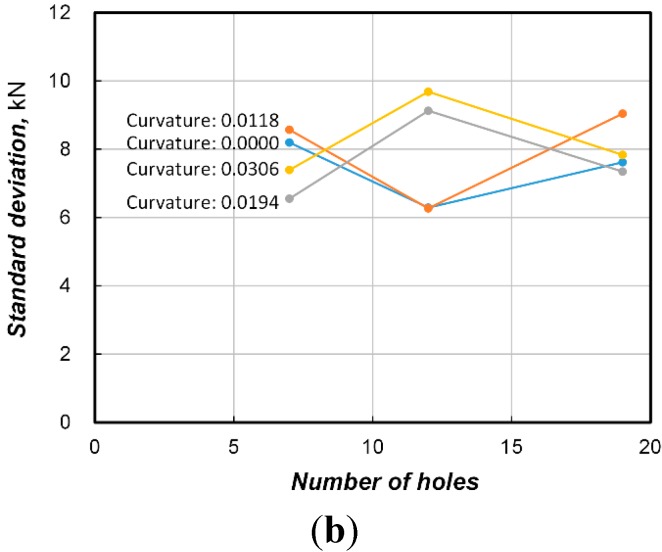
Pattern of standard deviation at final prestress stage. (**a**) Standard deviation of prestress force per number of strand according to change in sheath curvature; (**b**) Standard deviation of prestress force per curvature of sheath according to number of strand.

Figure 7 plots the patterns of the standard deviation at final prestress stage in order to observe the effect of the number of strands and curvature of the sheath on the distribution of the prestress force. In Figure 7a displaying the variation of the standard deviation per number of strands with respect to the increase of the curvature of the sheath, the standard deviation is seen to increase for the multi-strand system with the 12-hole anchor head but does not show any particular pattern for the cases with 7-hole and 19-hole anchor heads. A similar observation can also be made in Figure 7b that plots the standard deviation per curvature of sheath according to the number of holes. This indicates that the standard deviation of the prestress force is not influenced sensitively by the number of holes or the curvature of the sheath.

## 4. Discussion

### 4.1. Check for Normality 

**Figure 8:**
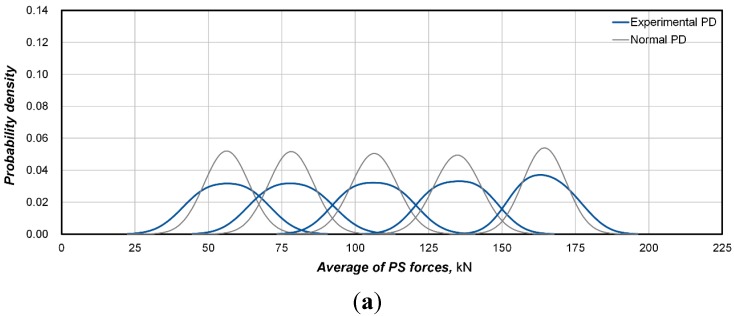

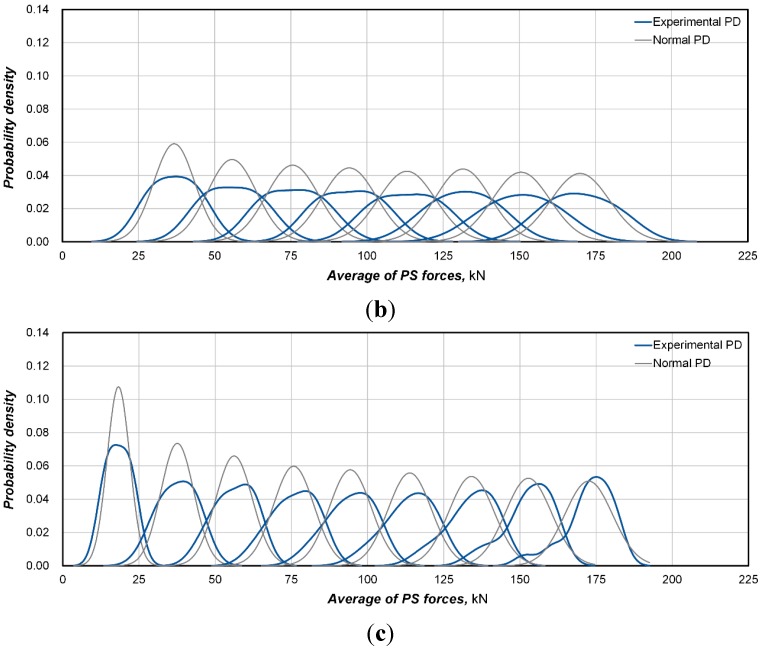
Comparison of the probability density functions of the prestress force with the normal distribution. (**a**) 7-hole system (specimen 7-1); (**b**) 12-hole system (specimen 12-1); (**c**) 19-hole system (specimen 19-1).

In view of the analysis of the test results in the previous section, the distribution of the prestress force was seen to exhibit some symmetry with respect to the average prestress force. Accordingly, it seemed of interest to check if the prestress force follows a normal distribution. To that end, the probability density functions described by the prestress force of the specimens at each prestress stage were compared with the normal distribution (Figure 8). The normal distribution was established using the average and standard deviation of the prestress force at each prestress stage. In addition, the probability density function of the prestress force is obtained by applying the smoothing technique [10] to prevent the problem caused by the derivation of different distributions according to the length of the intervals for the histogram. Even if the so-obtained probability density functions are seen to exhibit relatively small probability density at proximity of the average in the normal distribution, these functions fit very closely to the normal distribution. Accordingly, the probability density functions of the prestress force may be assumed as normal. The same observations can be drawn for the specimens that are not displayed in the figures.

To confirm the validity of such an assumption, the normality of the distribution is checked for a significance level of 5%. For this purpose, the Lilliefors method [11] is applied to cope with the small number of data acquired from the tests. The check for normality relative to the prestress force distribution of the specimens at each prestressing stage reveals that all the test data show a significance level above 5%, except for specimen 19-1 for which a significance level smaller than 5% is observed for one prestressing stage. This validates the assumption of normal distribution for the prestress force of the strands in the multi-strand system.

### 4.2. Derivation of Prestress Force Distribution Curve

The normal distribution is defined by the mean value and the standard deviation. Here, the average prestress force can be computed by dividing the actual total prestress force measured during the prestressing process by the number of strands. Therefore, the distribution curve of the prestress force can be obtained for a given relationship between the average and standard deviation of the prestress force.

**Figure 9:**
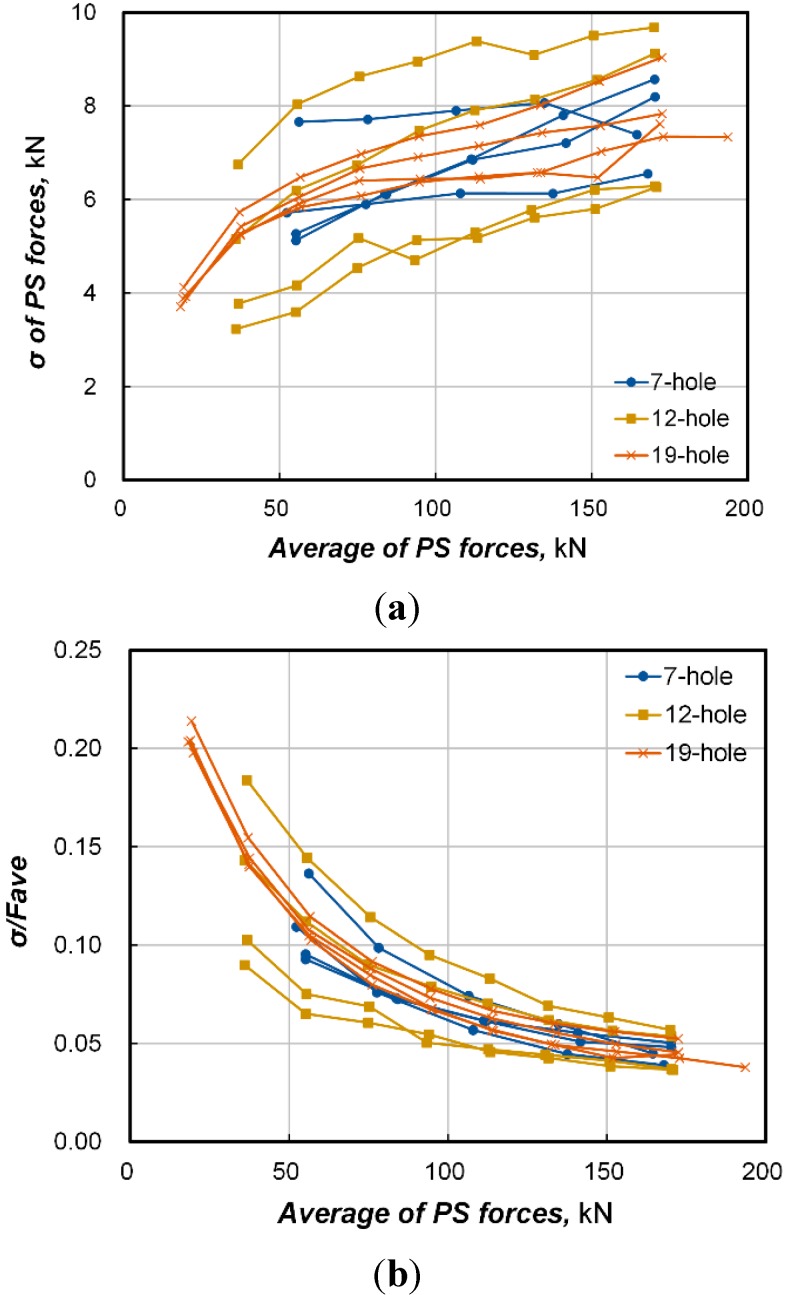
Fluctuation of standard deviation and coefficient of variation wrt average prestress force. (**a**) Standard deviation; (**b**) Coefficient of variation.

In Figure 7, we verified that the standard deviation of the prestress force is unrelated to the number of strands and curvature of the sheath. Accordingly, the standard deviation of the prestress force is likely to be dependent to the average prestress force only. Figure 9a plots the fluctuation of the standard deviation (σ) with respect to the average prestress force. It can be seen that the standard deviation tends on the whole to increase with the increase of the prestress force. Moreover, the standard deviation is also seen to have a wide range. Besides, Figure 9b, where the fluctuation of the coefficient of variation (COV) obtained by dividing the standard deviation by the average is plotted, shows that the fluctuation of the COV is reduced with a larger average prestress force but with a clear pattern. Consequently, it seems advisable to derive the relationship with the average prestress force using the COV rather than the standard deviation.

**Figure 10:**
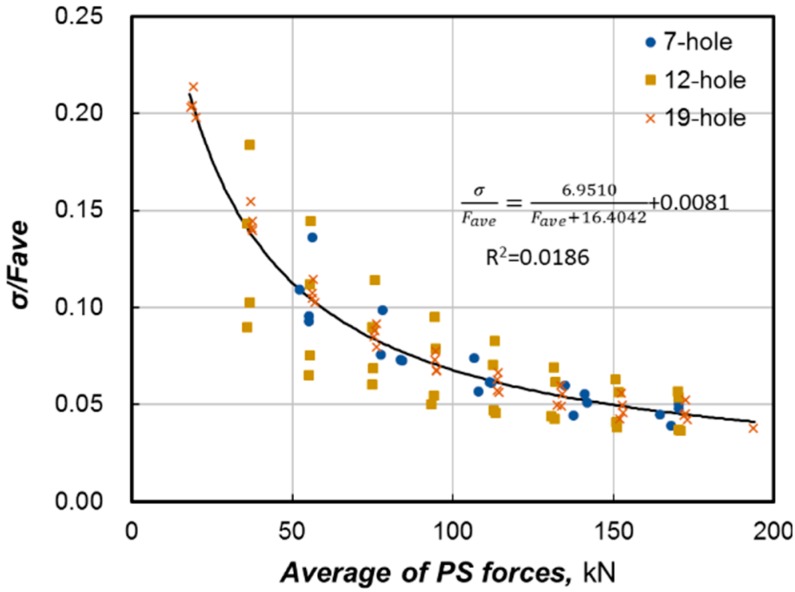
COV fitting curve.

Regression analysis was conducted to establish the relationship between the average and COV of the prestress force. Various types of functions were attempted and enabled to find out that the most suitable trend curve is the one expressed in Equation (1). Figure 10 concurrently plots the COV and fitting curve and reveals good agreement. This fitting curve will be complemented in the future with a larger number of data:

(1) $$\frac{\text{σ}}{F_{ave}}=\frac{6.9510}{F_{ave}+16.4042}+0.0081$$

### 4.3. Estimation of Changes in Prestress Force Distribution

The distribution pattern of the prestress force in the multi-strand system could be estimated by means of the observation of the test data. Considering that one strand among those in the multi-strand system is replaced by a smart strand, it is now necessary to propose a solution enabling to estimate the distribution pattern of the prestress force in the multi-strand system using the measurement provided by the single smart strand. 

**Figure 11:**
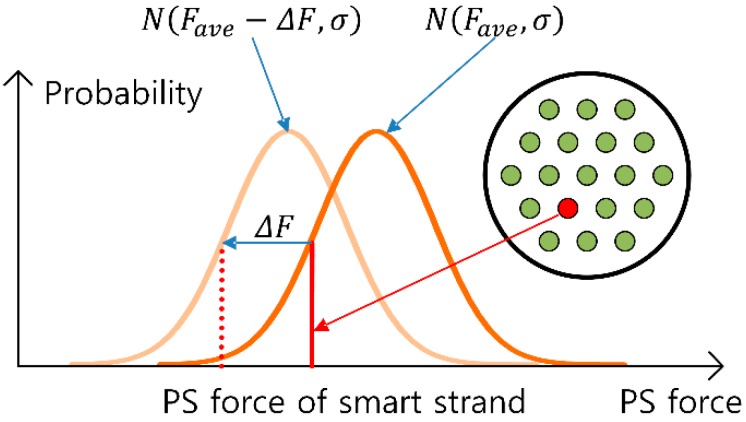
Estimation method of the prestress force.

The overall prestress force can be known by means of the hydraulic jack used throughout the prestressing process of the multi-strand system. Dividing this total prestress force by the number of strands gives the average prestress force from which the standard deviation can be computed using Equation (1). Figure 11 plots the so-obtained distribution curve of the prestress force. At this point of time, the measurement given by the smart strand represents only one value in the overall distribution of the prestress force and is likely to be different to the average prestress force.

The distribution pattern of the prestress force varies continuously all along the operation of the PSC structure. Therefore, the measurement given by the smart strand also varies and can be used as an indicator for the estimation of the variation in the distribution of the prestress force. Let ΔF be the change in the prestress force occurring in the smart strand from the start of prestress to a given time, it can be assumed reasonably that the distribution curve of the prestress force will also experience a shift by ΔF. As a matter of fact, the change in the distribution of the prestress force can be computed more accurately considering the deformation occurring within the cross-section of the structure but such process is meaningless since the distribution pattern of the prestress force is approximated as a normal distribution. Accordingly, the average prestress force at the changing time becomes $F_{ave}-\Delta F$ and the distribution curve of the prestress force can be estimated to be $N\left(F_{ave}-\Delta F,\;\text{σ}\right)$ recalling that the corresponding standard deviation does not experience any change during that period of time (Figure 11).

This method for the estimation of the prestress force distribution is applicable only for sound structures. The proposed method cannot be applied for structures that have experienced severe damage like the rupture of a wire since the distribution will not agree with that presented above. However, in such a case, the damage would be detected through a sudden and large variation of the measurement given by the smart strand.

## 5. Conclusions

The distribution characteristics of the prestress force in a multi-strand system have been obtained and used to propose a method for the estimation of the change in the prestress force distribution by exploiting the measurement provided by the a smart strand. Twelve types of multi-strand systems with different number of strands and sheath curvatures were considered and tested to measure the distribution of the prestress force by means of elastomagnetic (EM) sensors. The measurements enabled us to assume a normal distribution for the prestress force in each specimen. In the normal distribution, the average could be easily obtained by dividing the total prestress force by the number of strands. After verifying that the standard deviation has no particular sensitivity to the number of strands and curvature of sheath, a model expressing the standard deviation as a function of the average prestress force was proposed. The so-obtained average and standard deviation of the prestress force were then used to establish the distribution curve of the prestress force at prestressing stage. Thereafter, the change in the prestress force distribution occurring during the operation of the PSC structure can be estimated by shifting the distribution to an extent corresponding to the change in the prestress force measured by the smart strand. This method for the estimation of the prestress force distribution is believed to be exploitable for the management and maintenance of the prestress force as a critical factor in terms of the serviceability and safety of prestressed concrete structures.
